# Re-Evaluation of Binding Properties of Recombinant Lymphocyte Receptors NKR-P1A and CD69 to Chemically Synthesized Glycans and Peptides

**DOI:** 10.3390/ijms15011271

**Published:** 2014-01-17

**Authors:** Daniel Rozbeský, Jana Krejzová, Karel Křenek, Jan Prchal, Richard Hrabal, Milan Kožíšek, Lenka Weignerová, Michele Fiore, Pascal Dumy, Olivier Renaudet, Vladimír Křen

**Affiliations:** 1 Institute of Microbiology, Academy of Sciences of the Czech Republic, Vídeňská 1083, Prague 4 CZ14220, Czech Republic; E-Mails: rozbesky@gmail.com (D.R.); hoficek@centrum.cz (J.K.); krenek@biomed.cas.cz (K.K.); lenka.weignerova@gmail.com (L.W.); 2 Department of Biochemistry, Faculty of Science, Charles University in Prague, Hlavova 8, Prague 2 CZ12840, Czech Republic; 3 Department of Biochemistry, Institute of Chemical Technology in Prague, Technická 5, Prague 6 CZ16600, Czech Republic; 4 NMR Laboratory, Institute of Chemical Technology in Prague, Technická 5, Prague 6 CZ16600, Czech Republic; E-Mails: jan.prchal@vscht.cz (J.P.); richard.hrabal@vscht.cz (R.H.); 5 Gilead Sciences and IOCB Research Centre, Institute of Organic Chemistry and Biochemistry, Academy of Sciences of the Czech Republic, Flemingovo n. 2, Prague 6 CZ16610, Czech Republic; E-Mail: midlan@seznam.cz; 6 Département de Chimie Moléculaire—UMR CNRS 5250, Université Joseph Fourier, 570 rue de la chimie—BP 53, Grenoble cedex 9 F38041, France; E-Mails: michele.fiore@ujf-grenoble.fr (M.F.); pascal.dumy@ujf-grenoble.fr (P.D.); olivier.renaudet@ujf-grenoble.fr (O.R.)

**Keywords:** CD69, NKR-P1A, NMR titration, calorimetry

## Abstract

The binding of monosaccharides and short peptides to lymphocyte receptors (human CD69 and rat NKR-P1A) was first reported in 1994 and then in a number of subsequent publications. Based on this observation, numerous potentially high-affinity saccharide ligands have been synthesized over the last two decades in order to utilize their potential in antitumor therapy. Due to significant inconsistencies in their reported binding properties, we decided to re-examine the interaction between multiple ligands and CD69 or NKR-P1A. Using NMR titration and isothermal titration calorimetry we were unable to detect the binding of the tested ligands such as *N*-acetyl-d-hexosamines and oligopeptides to both receptors, which contradicts the previous observations published in more than twenty papers over the last fifteen years.

## 1. Introduction

Human CD69 and rat NKR-P1A are important lymphocyte receptors expressed on the surface of natural killer (NK) cells that play an important role in antiviral and antitumor immunity [1,2]. The first report on carbohydrate binding by NKR-P1A was published in 1994 by Bezouška *et al*. [3]. Binding and inhibition studies using neoglycoproteins indicated that NKR-P1A was a lectin with the following order of preference: GalNAc > GlcNAc >> Fuc >> Gal >Man. Its carbohydrate binding activity was also reported to be dependent on Ca^2+^, which was associated with the protein [3]. The identification of high-affinity ligands for NKR-P1A with significant potential for antitumor therapy was also described in a subsequent publication by Bezouska *et al*. [4]. The highest affinity ligands of the investigated carbohydrates, with *IC*_50_ values in the range 10^−9^–10^−12^ M, were oligosaccharides derived from heparin, oligosaccharide sequences of the blood group family, the ganglio family and glycosaminoglycans [4].

With CD69, the first indication of carbohydrate-specific binding was demonstrated in 1995 with GlcNAc and GalNAc as the most effective ligands of the investigated monosaccharides, and the presence of Ca^2+^ was required for carbohydrate binding [5].

Re-evaluations of carbohydrate binding activity for NKR-P1A [6] and CD69 [7] were published in 1999, in which some former co-workers reported that they observed no binding signals to any of the monosaccharides, in contrast to previously reported data [3–5]. In addition to this, a weak binding signal for the sulphated polysaccharide fucoidan was detected with CD69, however, this observation could be explained as an electrostatic interaction between the sulphated fucoidan and the His-tag sequence on the recombinant CD69 [7].

Research by K. Bezouška’s group then focused on aminosugars and their derivatives, because GlcNAc and GalNAc seemed to be effective ligands of both receptors. Binding experiments with a series of chitooligomers led to findings that chitotetraose is the best inhibitor of NKR-P1A [8,9]. A higher affinity was observed with simple derivates of chitooligomers, modified either using the epimerization of GlcNAc into ManNAc [10] or by replacing the NHAc group with OH [11]. Subsequent studies with different kinds of highly branched GlcNAc-terminated glycoclusters, such as GlcNAc-coated octavalent glycodendrimers (PAMAM-GlcNAc_8_), suggested high affinity for NKR-P1A [12–15].

Inhibition and equilibrium dialysis assays with CD69 revealed three separate binding sites for GlcNAc, two sites for GalNAc and further evidence that Ca^2+^ is an integral component of this receptor’s participation in carbohydrate binding [16], although no bound Ca^2+^ had been observed in the crystal structure of CD69 [17]. Later experiments suggested that the binding of GlcNAc to dimeric CD69 proceeded in a cooperative fashion with *K*_d_ = 0.4 μM, which was estimated using NMR titration in a buffer without calcium [18]. This observation contradicted previous reports that the presence of Ca^2+^ in the buffer during analysis is essential [16]. In later studies, one of the tested branched *N*-acetyl-d-hexosamines prepared synthetically exhibited *K*_d_ = 3.4 nM, indicating significant potential as a glycomimetic in antitumor therapy [19]. None of above compounds have been patented and no clinical trials have been started.

Several classes of compounds have been recently synthesized in order to identify other high-affinity ligands for NKR-P1A and CD69. Both receptors appeared to have extraordinary affinity for certain disaccharides such as ManNAc(1→4)Glc [20] or deoxynorijimycin and its hexosaminyl derivates [21] as well as negatively charged oligosaccharides and glycosides [22–24]. Monovalent and bivalent LacdiNAc glycomimetics prepared via enzymatic synthesis were also found to be efficient NK cell activators [25,26]. In addition, carboxylated calixarenes [27], glycosyl 1,2,3-triazoles [28] and glycoconjugates of the LELTE peptide [29] have been reported as new prominent classes of high-affinity ligands. The identification of the LELTE pentapeptide as a high-affinity ligand for CD69 in JACS [29] is referred to in a conference abstract from the 11th International Congress of Immunology, which was held in Stockholm in 2001. Surprisingly, there is no mention of the binding of the LELTE peptide or other peptides in the conference abstract [30]. The dimerized heptapeptide LELTEGY, a putative strong ligand of CD69, was recently reported to exhibit remarkable antimelanoma activity [31].

Due to significant inconsistencies in the reports, both published and unpublished, about the binding of saccharides and peptides to both receptors, we re-evaluated the binding of several saccharides and the LELTE peptide to recombinant CD69 and NKR-P1A using NMR titration and isothermal titration calorimetry. Our analyses do not support the previously reported data on the specific binding of NKR-P1A and CD69 to the tested ligands.

## 2. Results and Discussion

### 2.1. Results

#### 2.1.1. Protein Expression, Refolding and Purification

**Figure 1.**
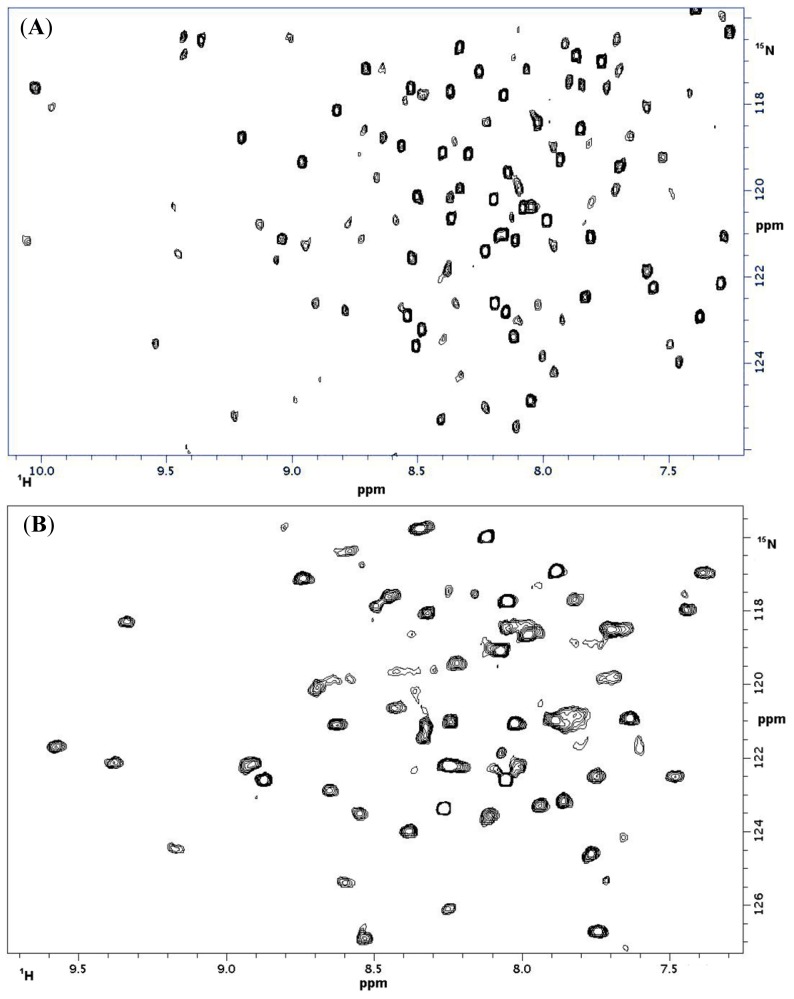

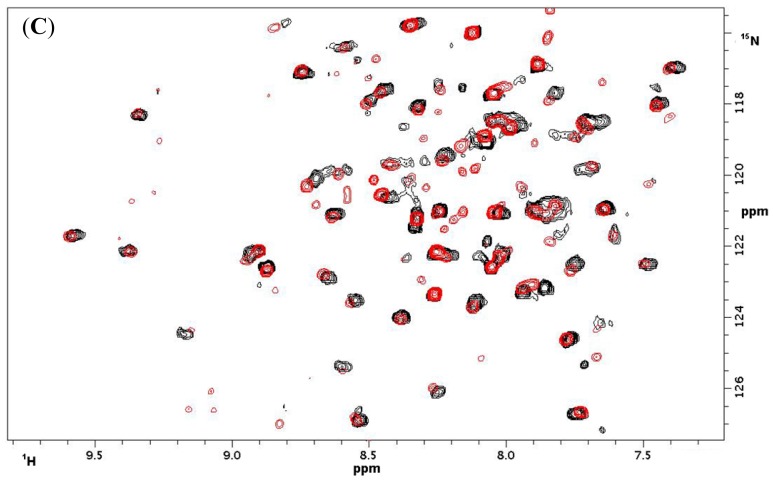
(**A**) NMR spectrum of rat NKR-P1A; (**B**) NMR spectrum of human CD69; (**C**) NMR spectrum of human CD69 compared with human CD69 from Brno [32].

Parts of the extracellular domains of the CD69 encompassing residues G70-K199 and NKR-P1A-encompassing residues A90-K215 were expressed in *E. coli*. After the induction of protein expression, both proteins precipitated into inclusion bodies, from which they could be easily refolded *in vitro*. The purification of recombinant proteins was done using a combination of ion exchange chromatography and gel filtration. Before NMR and ITC titration, the identity of the prepared protein samples was verified by mass spectrometry. Monodispersity and high purity was further confirmed by measuring the ^1^H-^15^N HSQC spectra of uniformly ^15^N labeled proteins (Figure 1A,B). The ^1^H-^15^N HSQC spectra indicated good dispersion of the backbone and side chain signals with a good chemical shift dispersion in the proton dimension, indicating a compact fold of both proteins. The superposition (Figure 1C) of the ^1^H-^15^N HSQC spectrum of CD69 onto the one acquired previously showed identical folds of the CD69 prepared by ourselves and the protein described previously [32]. The ^1^H-^15^N HSQC of rat NKR-P1A has not yet been published.

#### 2.1.2. Isothermal Titration Calorimetry Measurements

**Figure 2.**
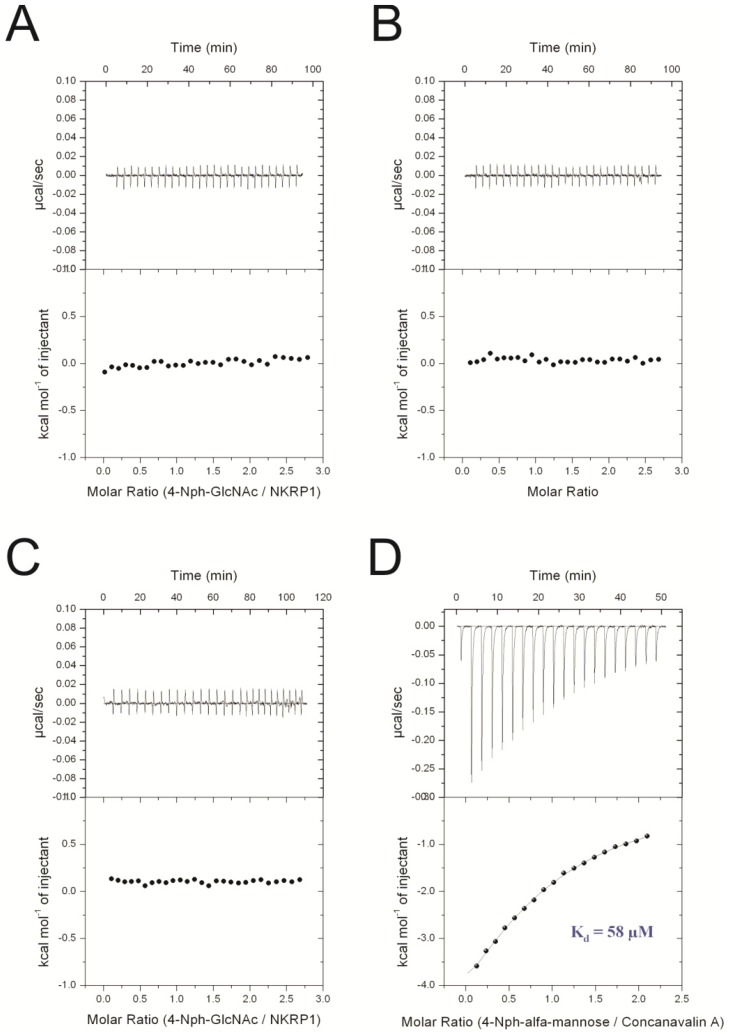
Isothermal titration of rat NKR-P1A with *p*NP-GlcNAc (**A**) and a corresponding control dilution in which *p*NP-GlcNAc was injected into a sample cell containing buffer alone (**B**); Microcalorimetry titration of rat NKR-P1A was also performed with *p*NP-GlcNAc supplemented with 10 mM CaCl_2_ (**C**) due to potential calcium-dependent binding; Isothermal titration of *p*NP-α-mannose with Concanavalin A was used as a positive control (**D**).

Microcalorimetry titrations of mannose, GlcNAc, chitobiose, *p*NP-GlcNAc and the LELTE peptide with NKR-P1A and CD69 were performed at 25 °C using a VP-ITC system (MicroCal, GE Healthcare, Pittsbugh, USA). All these titrations were accompanied by control dilutions in which saccharides were injected into the sample cell containing only the buffer. The titration of *p*NP-GlcNAc into the NKR-P1A protein and corresponding control dilution is shown in Figure 2A,B. Since the saccharide binding of NKR-P1A could be Ca^2+^-dependent, *p*NP-GlcNAc was titrated into NKR-P1A supplemented with 10 mM CaCl_2_ (Figure 2C). The results of the other titrations are summarized in Supplementary Figures S1 and S2. In all the performed experiments, the heats released by the titrations of the saccharides and the LELTE peptide into NKR-P1A and CD69 did not differ from the saccharide dilution energies. This observation enables us to conclude that there was no interaction identified by isothermal titration calorimetry. The microcalorimetry titration of *p*NP-GlcNAc with Concanavalin A (Sigma-Aldrich, Prague, Czech Republic) was used as a positive control (Figure 2D) and the calculated *K*_d_ of this interaction was 58 μM.

#### 2.1.3. NMR Titrations

**Figure 3.**
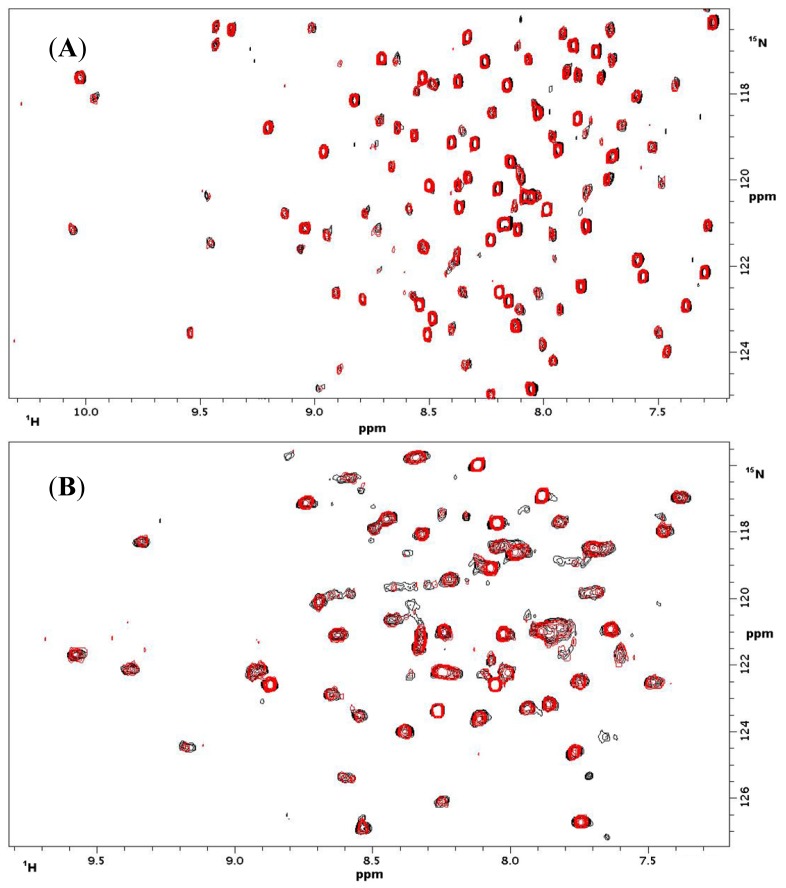
(**A**) Superposition of NMR spectra of rat NKR-P1A with (red) and without (black) *p*NP-GlcNAc; (**B**) Superposition of NMR spectrum of human CD69 with (red) and without (black) *p*NP-GlcNAc.

NMR titrations of mannose, *p*NP-GlcNAc, 1,2-*bis*[*N*-(2-acetamido-2-deoxy-β-d-glucopyranosyl)- thioureido]-decane [26] and the LELTE peptide with NKR-P1A and CD69 were performed at 25 °C in molar ratios of 1:1 and 1:2 (protein:ligand). In all cases the ^1^H-^15^N HSQC spectra were measured and compared with the ^1^H-^15^N HSQC spectrum of the protein without the ligand. The superposition of ^1^H-^15^N HSQC spectra with and without *p*NP-GlcNAc (Figure 3) revealed no significant differences in their peak positions and relative intensities (Table 1). The superpositions of spectra with other ligands (Supplementary Figure S3) were almost identical, which indicated no interaction between the tested ligands and NKR-P1A or CD69.

### 2.2. Discussion

Due to serious discrepancies in the published data on the binding properties of human CD69 and rat NKR-P1A to oligosacharides and peptides, we decided to thoroughly re-examine their binding activity to selected ligands. Both proteins have been previously shown to bind a wide range of saccharides as well as peptides, carboxylated calixarenes and glycosyltriazoles, indicating significant potential in antitumor therapy. Besides the ligands described by Bezouška’s group, no other small-molecule ligand for human CD69 and rat NKR-P1A has been reported so far, but the protein Clr11 was recently observed to bind rat NKR-P1A [33]. In the previous articles by Bezouška and coworkers, the binding activity was mainly documented using plate binding assays with the proteins labeled with fluorescent or radioactive probes. In a few publications, equilibrium dialysis and NMR titrations were performed by Bezouška’s co-workers [18,19], however during the NMR titrations, only the signal of the free saccharide was analyzed in a 1D spectrum. Therefore, we decided to investigate the receptor-ligand interaction using more accurate techniques such as isothermal titration calorimetry and NMR titrations, monitoring the signals of the protein in ^1^H-^15^N HSQC spectra. Since the majority of the described ligands were derivatives of GlcNAc or the LELTE peptide, we analyzed the interaction between CD69 or NKR-P1A and ligands such as 1,2-bis[*N*-(2-acetamido-2-deoxy-β-d-glucopyranosyl)-thioureido]-decane, *p*NP-GlcNAc, the LELTE peptide and mannose. In calorimetric titrations, Concanavalin A was used as a positive control and D-mannose as a negative control.

The extracellular domains of CD69 and NKR-P1A were generated in a bacterial expression system according to the detailed protocols provided by Bezouška and co-workers. The protein identity was confirmed by mass spectrometry, amino acid analysis, and the protein fold was verified by good signal dispersion in ^1^H-^15^N HSQC spectra. The protein fold of CD69 was also the same as described in the above publication [32], which was demonstrated by the superposition of ^1^H-^15^N HSQC spectra.

Using calorimetric and NMR titrations, we failed to detect any binding of the tested ligands to human CD69 and rat NKR-P1A. For the analysis, the same buffers were used as in the previously published articles. Since the saccharide binding properties were reported to be Ca^2+^-dependent, we investigated the effect of Ca^2+^ on saccharide binding, however no binding was detected. We therefore conclude that the extracellular domains of human CD69 and rat NKR-P1A prepared by the provided protocols do not bind 1,2-*bis*[*N*-(2-acetamido-2-deoxy-β-d-glucopyranosyl)-thioureido]-decane, *p*NP-GlcNAc, mannose and the LELTE peptide. This observation contradicts the results published in the previous 25 publications by Bezouška’s group [3–5,8–16,18–31]. We suspect that the extracellular domains of human CD69 and rat NKR-P1A are also unlikely to bind the other ligands described in the previous papers, because the majority of them are derivates of GlcNAc or the LELTE peptide.

In addition to this, the recombinant CD69 used in our analyses is part of the extracellular domain encompassing part of the stalk region and the C-type lectin-like domain, whereas the recombinant NKR-P1A only encompasses the extracellular C-type lectin-like domain without the stalk. Additionally, recent binding experiments with a similar NKp30 receptor belonging to the immunoglobulin superfamily showed that the ligand-binding region is not sufficient for ligand recognition. In fact, the entire glycosylated stalk domain connected with the ligand binding region is crucial for ligand binding and subsequent signalization [34]. Therefore, we do not exclude the possible binding of other saccharides or glycoproteins to CD69 or NKR-P1A as well as saccharides or glycoproteins to other constructs of CD69 or NKR-P1A.

The lack of binding activity of CD69 and NKR-P1A to the tested ligands raises questions about the discrepancy between our findings and the 25 previously published publications. Additionally, two re-evaluations of the saccharide binding activity of NKR-P1A [6] and CD69 [7] were published by Bezouška’s former coworkers in 1999. In other articles by Karel Bezouška, several saccharides and the LELTE derivatives were shown to exhibit significant potential in antitumor therapy with nanomolar binding constants to CD69 [19,31].

## 3. Experimental Section

### 3.1. Protein Expression and Purification

The expression plasmid for human CD69 (residues G70-K199) in the pRSET B vector was provided by Daniel Kavan and the expression plasmid for rat NKR-P1A (residues A90-K215) in the pET-30a(+) vector was provided by K. Bezouška. Both expression plasmids were verified by DNA sequencing. For protein production, the expression plasmids were transformed into the *E. coli* BL21(DE3) Gold strain (Stratagene, Agilent Technologies, Santa Clara, CA, USA ). Large-scale protein production was carried out in 1 L of LB medium supplemented with antibiotics, whereas the ^15^N-labeled proteins were produced in 1 L of M9 minimal medium containing antibiotics and ^15^NH_4_Cl (Cambridge Isotope Laboratories, Tewksbury, MA, USA) as the sole nitrogen source. Protein expression was induced by adding isopropyl-β-d-thiogalactopyranoside to a final concentration of 1 mM, when the cell density reached *OD*_600_ = 0.8. After the induction of protein expression, cells were grown for 6 h at 37 °C with shaking at 220 rpm. The cells were then harvested by centrifugation at 6000 *g* for 10 min, and inclusion bodies were isolated [35]. The inclusion bodies of CD69 were solubilized in 2 mL of 50 mM Tris-HCl (pH 8.0), 6 M guanidine-HCl and 100 mM DTT, 1 mM leupeptin and 1 mM PMSF, whilst the inclusion bodies of NKR-P1A were solubilized in the same buffer apart from the 100 mM DTT, which was replaced with 10 mM DTT. Insoluble material was removed from the solution by centrifugation at 50,000 *g* for 30 min at 20 °C and the proteins were refolded *in vitro* by rapid dilution into a 100-fold excess of refolding buffer. The refolding buffer for CD69 contained 100 mM Tris-HCl (pH 7.8), 0.4 M L-Arg, 10 mM CaCl_2_, 4 mM cysteamine, 2 mM cystamine, 1 mM NaN_3_ and 0.1 mM PMSF, whereas the refolding buffer for NKR-P1A included 50 mM Tris-HCl (9.0), 1 M L-Arg, 100 mM CaCl_2_, 9 mM cysteamine, 3 mM cystamine, 1 mM NaN_3_ and 1 mM PMSF. The refolding mixture was incubated at 4 °C for 1 h and then dialyzed twice at 4 °C against 8 L of dialysis buffer for 6 h. Whilst NKR-P1A was dialyzed twice against the same buffer containing 15 mM Tris-HCl (pH 9.0), 9 mM NaCl and 1 mM NaN_3_, CD69 was dialyzed against a buffer including 50 mM Tris-HCl (pH 7.8), 0.5 M NaCl, 1 mM NaN_3_ and then against a buffer containing 20 mM sodium acetate (pH 5.5), 9 mM NaCl and 1 mM NaN_3_. The refolded proteins were concentrated by ultrafiltration using cellulose membranes (Millipore, Billerica, MA, USA) with a 10 kDa cut-off and then purified using ion exchange chromatography in a SP Sepharose FF column (GE Healthcare, Little Chalfont Bucks, UK) with CD69 or a Q Sepharose FF column (GE Healthcare, Little Chalfont Bucks, UK) with NKR-P1A. The proteins were eluted by a linear gradient of NaCl from 9 mM–1 M and fractions containing the folded proteins were pooled and concentrated by rapid ultrafiltration using centrifugal filter devices (Millipore, Billerica, MA, USA) with a 10 kDa cut-off. The concentrated proteins were finally purified by gel filtration in a Superdex 75 10/300 GL column (GE Healthcare, Little Chalfont Bucks, UK). Protein concentration was estimated by Bradford assay (Bio-Rad, Hercules, CA, USA) and finally measured using amino acid analysis. Before ITC and NMR titrations, the CD69 was dialyzed into a buffer containing 10 mM MES (pH 5.8), 49 mM NaCl and 1 mM NaN_3_, whereas the NKR-P1A was dialyzed into a buffer containing 20 mM Tris-HCl (pH 7.8), 150 mM NaCl and 1 mM NaN_3_.

### 3.2. Calorimetric Experiments

The binding of mannose, chitobiose and *p*NP-GlcNAc (Sigma-Aldrich), GlcNAc (GLYCON Biotech, Luckenwalde, DE) and the LELTE peptide to NKR-P1A and CD69 were monitored using a VP-ITC calorimeter (MicroCal Inc., GE Healthcare, Pittsbugh, USA) at 25 °C. Typically, 10 μL aliquots of 100 μM saccharide were injected stepwise into a sample cell containing 1.43 mL of 8 μM protein solution until saturation was achieved. All the experiments were accompanied by the corresponding control experiments where the putative ligands were injected into buffer alone. The signals from the titrations were analyzed using the software Origin 7.0 (GE Healthcare, Pittsbugh, USA developed by MicroCal. Microcalorimetry titration with Concanavalin A (Sigma-Aldrich, Prague, Czech Republic) was used as a positive control and mannose as a negative control.

### 3.3. NMR Titration Experiments

NMR data were measured in a Bruker Avance^III^ 600 MHz NMR spectrometer equipped with a cryoprobe (Bruker BioSpin, GmbH, Bremen, Germany) at a ^1^H working frequency of 600.13 MHz at 25 °C. Ligand binding was monitored by observing changes in the relative intensities and peak positions in ^1^H-^15^N HSQC experiments after titrating the NKR-P1A protein with a ligand. After protein and ligand mixing in a molar ratio of 1:1 and 1:2, the ^1^H-^15^N HSQC spectrum was measured and compared to the ^1^H-^15^N HSQC spectrum of the free protein. The data were processed and analyzed in Topspin 3.0 (Bruker BioSpin, GmbH, Germany).

## 4. Conclusions

Nobody with the exception of Prof. Karel Bezouška has been able to reproduce the saccharide binding of CD69 and NKR-P1A to date. In conclusion, our findings in combination with ethical considerations [36] concerning K. Bezouška raise serious doubts about the credibility of the binding properties of human CD69 and rat NKR-P1A to saccharide ligands and the LELTE peptide as described in the previous papers co-authored by K. Bezouška, which now need to be thoroughly revised (see also the “Press Release on Scientific Misconduct” in the Supplementary Material).

## Supplementary Information



## Figures and Tables

**Table 1.** Chemical shift differences of four representative pairs of corresponding cross-peaks (from different spectral regions), demonstrating that the chemical shift changes caused by the addition of the ligand (L = *p*NP-GlcNAc) were negligible. Differences on the order of 10^−3^ ppm cannot be ascribed to the ligand binding and result from experimental error.

CD 69		CD 69 + L		NKR-P1A		NKR-P1A + L	
			
^1^H (ppm)	^15^N (ppm)	^1^H (ppm)	^15^N (ppm)	^1^H (ppm)	^15^N (ppm)	^1^H (ppm)	^15^N (ppm)
9.5772	121.684	9.5784	121.648	10.0616	121.160	10.0579	121.1426
9.3354	118.332	9.3341	118.269	9.2078	118.775	9.2070	118.772
8.5472	123.488	8.5516	123.538	7.0786	117.172	7.0773	117.179
7.7638	124.592	7.7651	124.581	8.0078	123.829	8.0068	123.820

## Abbreviations

GalNAc: 2-acetamido-2-deoxy-d-galactopyranose
GlcNAc: 2-acetamido-2-deoxy- d-glucopyranose
*p*NP-GlcNAc: *p*-nitrophenyl 2-acetamido-β-d-glucopyranoside
ITC: isothermal titration calorimetry
ManNAc: 2-acetamido-d-mannopyranose
NK: natural killer
