# Supplementary material for: Re-Evaluation of Binding Properties of Recombinant Lymphocyte Receptors NKR-P1A and CD69 to Chemically Synthesized Glycans and Peptides
Source: Int J Mol Sci. 2014 Jan 17;15(1):1271–83. doi: 10.3390/ijms15011271 (PMC3907868; doi:10.3390/ijms15011271)
Supplement: Supplementary file 1 [file ijms-15-01271-s001.pdf]

Supplementary Information

Figure S1. Isothermal titration of rat NKR-P1A with GlcNAc (**A**); chitobiose (**C**); LELTE peptide (**E**) and mannose (**G**); The corresponding control dilutions in which ligand was injected into the sample cell containing buffer alone are shown in (**B**, **D**, **F** and **H**).

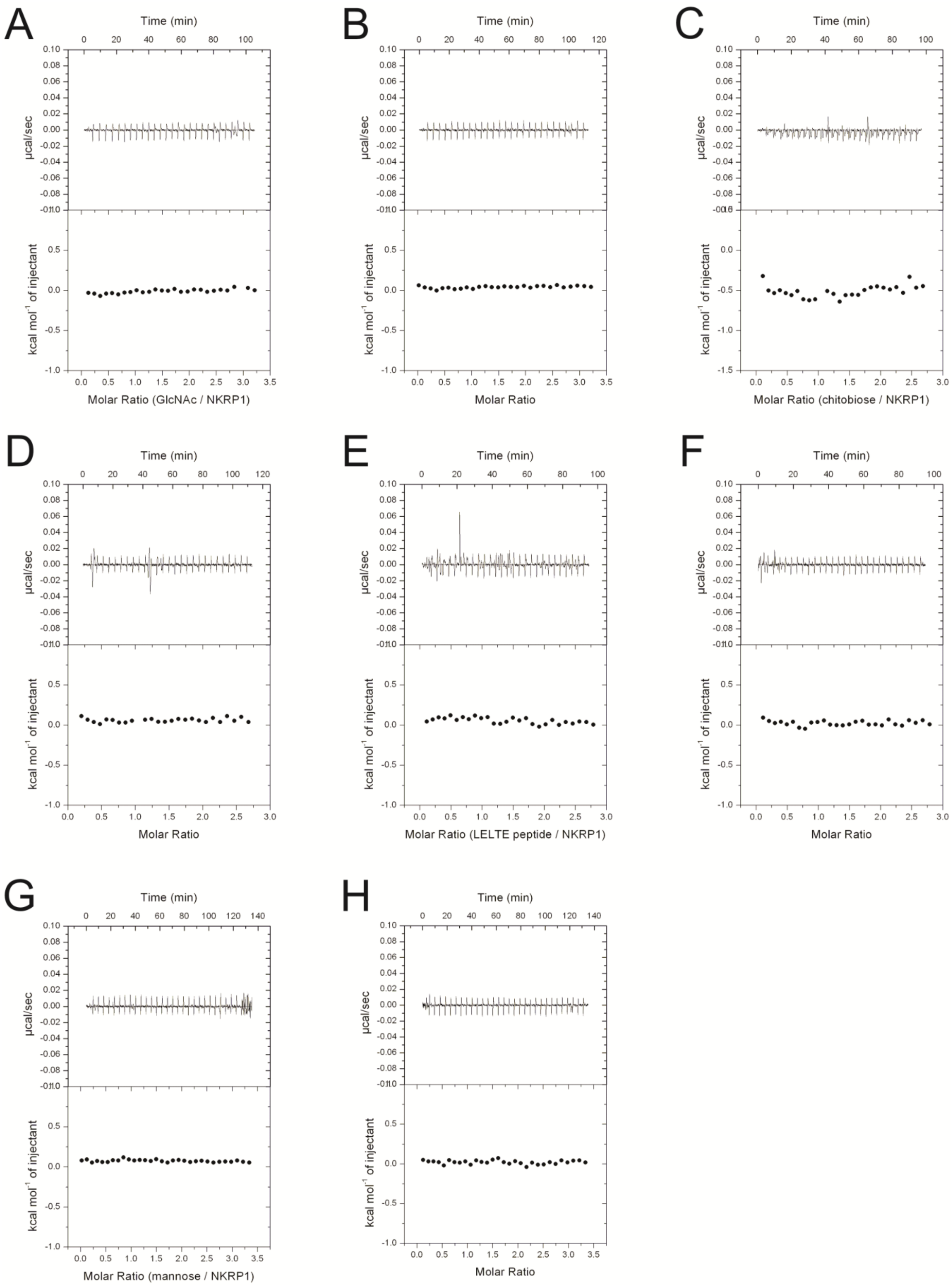

Figure S2. Isothermal titration of human CD69 with GlcNAc (**A**); *p*NP-GlcNAc (**C**); LELTE peptide (**E**) and mannose (**G**); The corresponding control dilutions in which ligand was injected into the sample cell containing buffer alone are shown in (**B**, **D**, **F** and **H**).

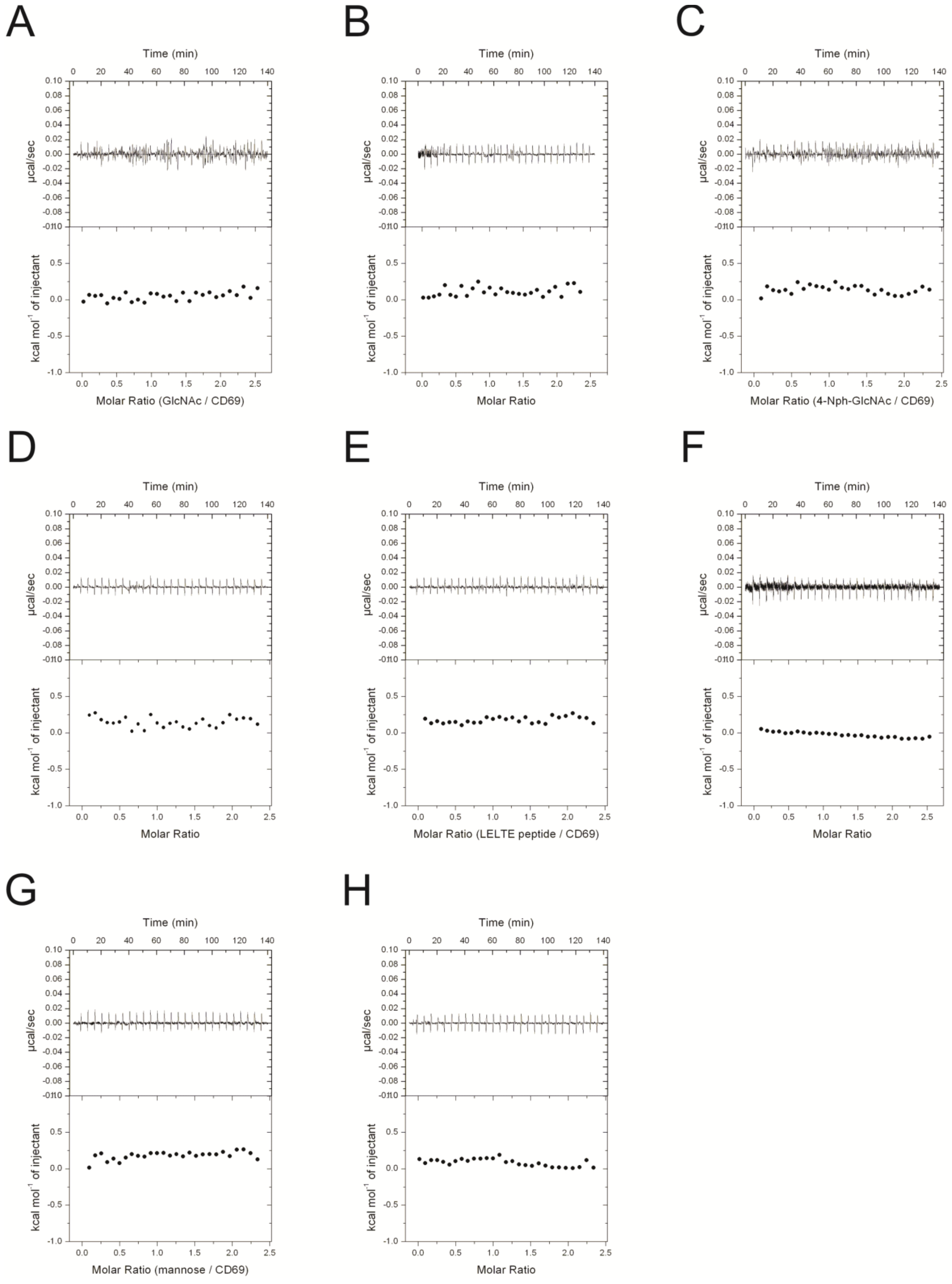

Figure S3. (**A1**) Superposition of NMR spectra of rat NKR-P1A with (red) and without (black) mannose; (**A2**) Superposition of NMR spectra of rat NKR-P1A with (red) and without (black) the LELTE; (**A3**) Superposition of NMR spectrum of human CD69 with (red) and without (black) 1,2-*bis*[*N*-(2-acetamido-2-deoxy-β-D-glucopyranosyl)-thioureido]-decane; (**B1**) Superposition of NMR spectrum of human CD69 with (red) and without (black) mannose; (**B2**) Superposition of NMR spectrum of human CD69 with (red) and without (black) the LELTE; (**B3**) Superposition of NMR spectrum of human CD69 with (red) and without (black)1,2-*bis*[*N*-(2-acetamido-2-deoxy-β-D-glucopyranosyl)-thioureido]-decane.

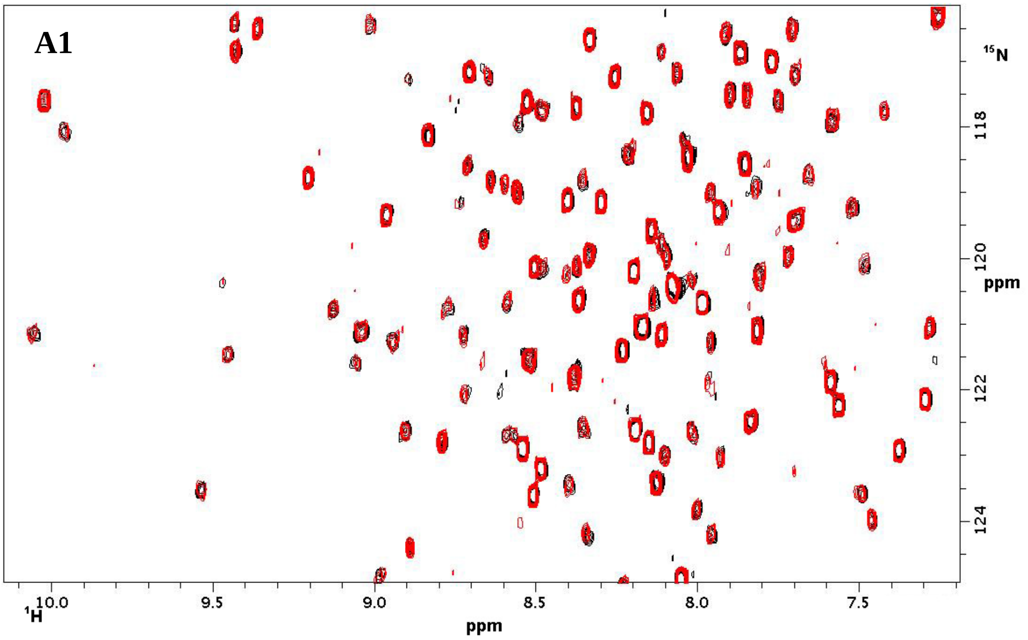

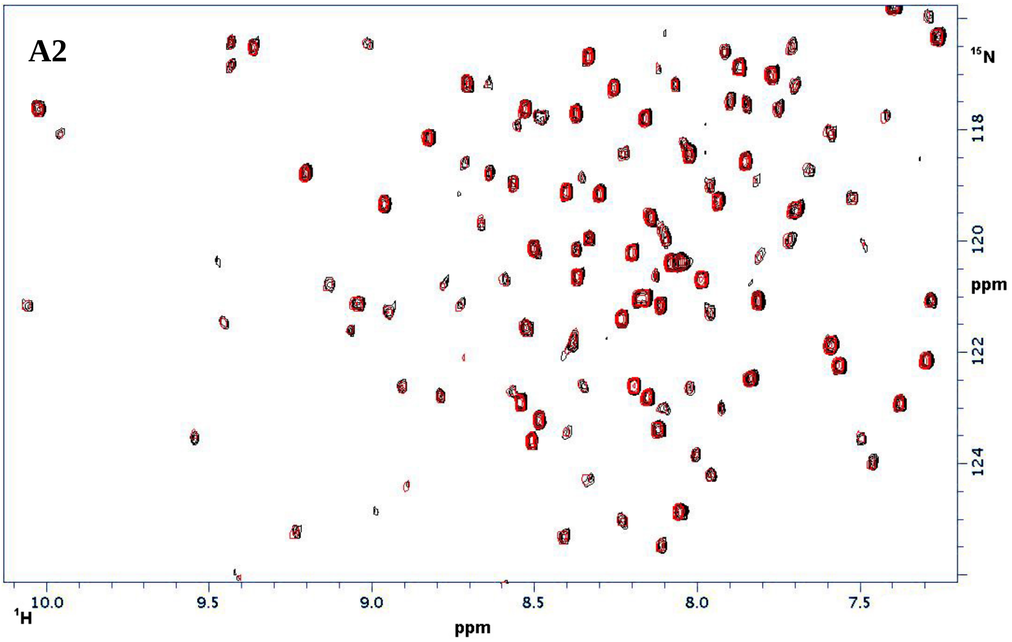

Figure S3. *Cont.*

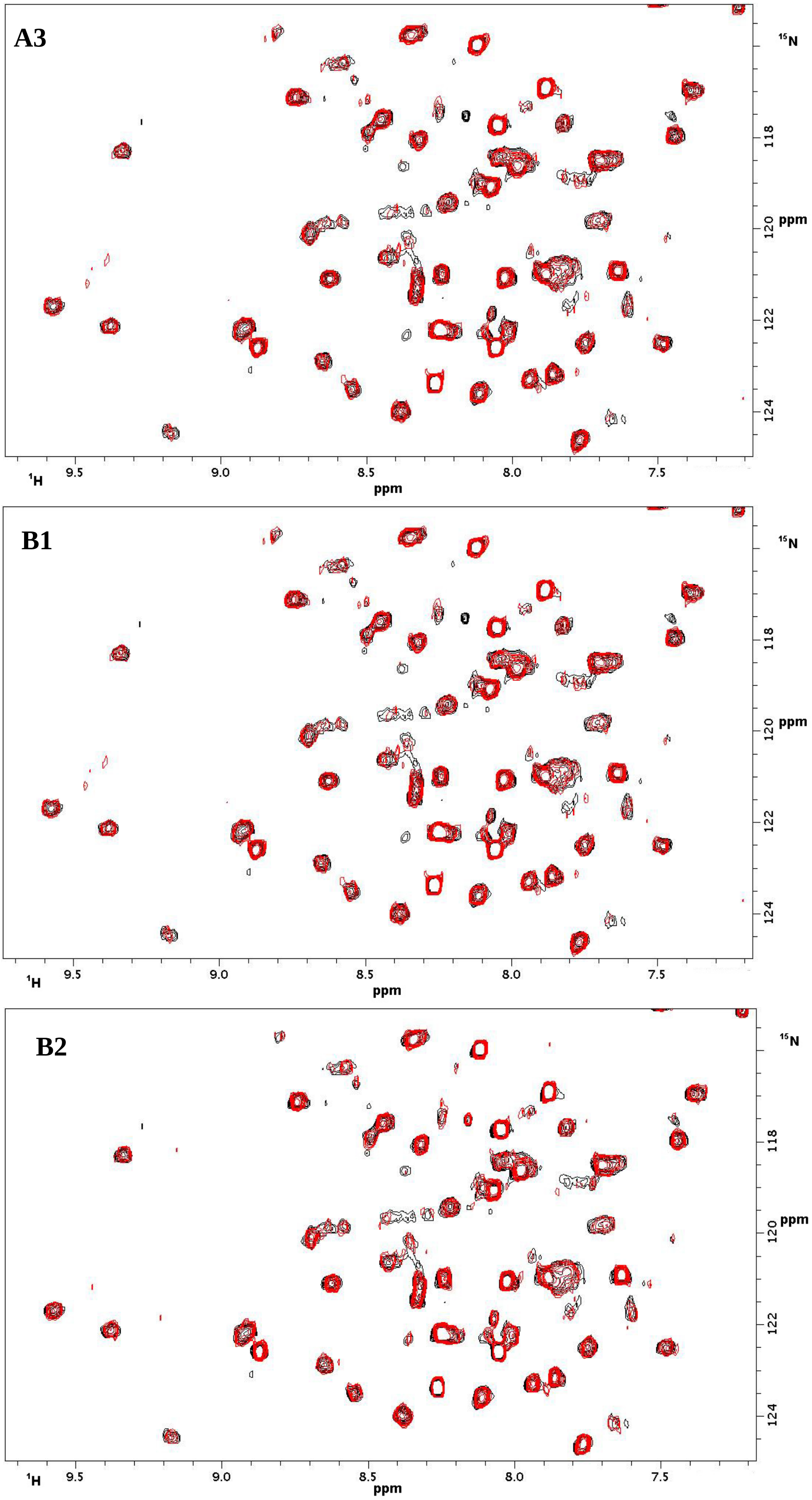

Figure S3. *Cont.*

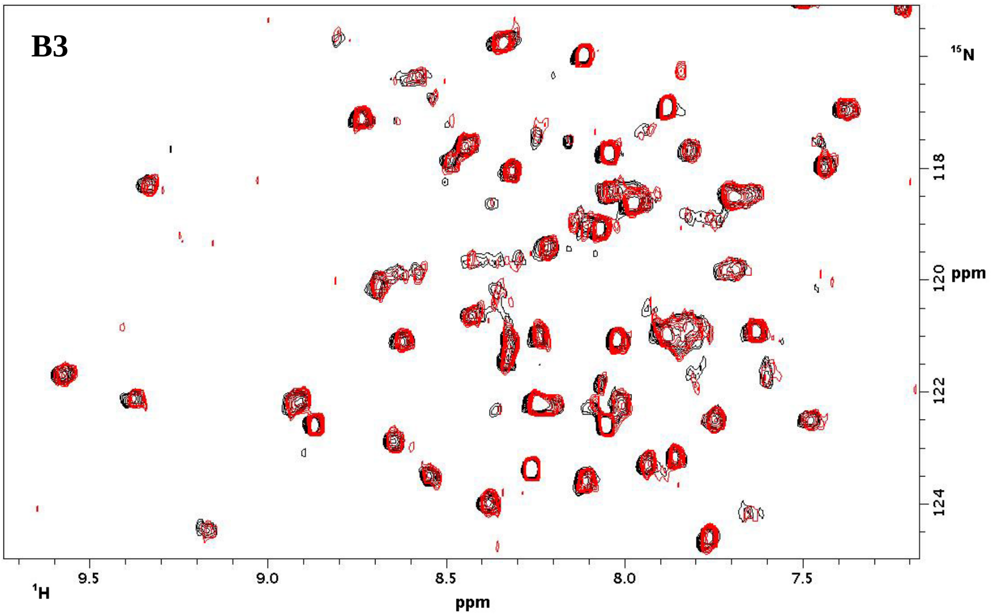

For the General Public

Press Release on Scientific Misconduct

The Institute of Microbiology AV ČR, v. v. i., and Faculty of Sciences of the Charles University in Prague, on the basis of the report of the Ethics Commission regretfully announce that in recent years Prof. RNDr. Karel Bezouška, DSc. has most likely repeatedly committed scientific misconduct (*i.e.*, breach of ethics of scientific work). Both institutions have terminated the employment of Prof. Bezouška, and the coauthors of all the respective papers have been asked to review all experiments and their interpretations.

The Ethics Committee was composed of:

Doc. RNDr. Jan Černý, Ph.D., Faculty of Science, Charles University in Prague

RNDr. Jiri Gabriel, PhD., Institute of Microbiology

Prof. RNDr. Helena Illnerová, DSc., Academy of Sciences

Prof. RNDr. Stanislav Komárek, Dr., Faculty of Science, Charles University in Prague

Prof. Ing. Karel Štulík, PhD., Faculty of Science, Charles University in Prague

Contact person: Doc. Dr. Jan Černý, coordinator of the Ethics Committee, Faculty of Science, Charles University in Prague. E-mail: jan.cerny@natur.cuni.cz

Below is a summary of the report of the Ethics Commission, which was set up by the Dean of the Faculty of the Charles University and Director of the Institute of Microbiology AV Czech Republic (ČR), to identify and assess whether Prof. RNDr. Karel Bezouška, DSc. had committed scientific misconduct during his scientific career.

The Ethics Commission on the basis of their findings declares that Prof. RNDr. Karel Bezouška, DSc. with the highest probability committed "scientific misconduct or dangerous or irresponsible deviations from accepted practice in how to perform research". This scientific misconduct occurred repeatedly. It is considered a great pity, as Prof. Bezouška was considered an excellent biochemist, one

of the founders of Proteomics in the Czech Republic, as well as a good lecturer and dedicated worker. The publishing activities of Prof. Bezouška consisted of over 100 articles in peer-reviewed international journals. Although the articles included a significant quantity of high-quality experimental work, unfortunately, in some cases they contained data that now cannot be reproduced.

The opinion of the British Medical Research Council (MRC) in 1999 was considered of particular value and importance for the Commission. The MRC on the initiative of Prof. T. Feizi dealt with the publications produced in the years 1993–1995 in collaborations with the Glycosciences Laboratory in Harrow (UK), of which Prof. Feizi was the head, together with a Laboratory of the Institute of Microbiology, ČSAV, in which Dr. Karel Bezouška worked. The group of Prof. Feizi was then unable to repeat the results of Dr. Bezouška experimentally. An investigative Commission of the MRC found in one of the four papers irregularities, which Dr. Bezouška could not explain satisfactorily. These facts led the MRC to suspect that primary data had been modified and also that the experimental samples had been contaminated by other proteins. The Commission also expressed deep concern over the fact that the works were in several cases not reproducible or apparently only the results from just one experiment, which later failed to be reproducible, were included in the papers. The Commission concluded that Dr. Bezouška committed scientific misconduct. In 1999 the report was received by Dr. Bezouška, the head of the group in Prague (deceased) and one worker (lady) of the group, together with the request of the Director General of MRC, to inform the Director of the Academy of Medical Sciences in Prague. The recipients of the report informed neither the President of the Academy, nor the head of the Institute of Microbiology AV ČR nor the head of the Faculty of Sciences, Charles University. The existence of this report remained unknown until 2012, when the Ethics Committee investigated the case. If this case had been investigated by the Czech side, it would probably have prevented further repetition of the scientific misconduct by Prof. Bezouška.

In 2012 three students from the laboratory of the Institute of Microbiology, with which Prof. Bezouška cooperated, became suspicious of manipulation of experimental material while testing the binding of carbohydrates to NKR-P1 protein. The reason for this was data inconsistencies, which in their opinion, were not possible to explain without external intervention. One of the students carried out verification tests, which revealed that the experimental material stored in the fridge had been manipulated by somebody without the knowledge of the laboratory workers. After this, a camera system was installed in the room immediately, allowing monitoring of the fridge. Cameras revealed that on the night of the 19/20.3. Prof. Bezouška on two occasions entered the room and on 21.3. again entered the room and manipulated the experimental material in the fridge. The Ethics Commission, after hearing all the witnesses, including the hearing of Prof. Bezouška, concluded that Prof. Bezouška had tampered with the samples and most probably repeatedly fabricated the positive results, so that they would confirm the binding of carbohydrates to the protein NKR-P1. The positive results reported by Prof. Bezouška on the binding of the carbohydrates to protein NKR-P1 repeatedly failed to be repeated or reproduced by additional independent laboratories.

The Ethics Commission studied a large amount of information including work, in which Prof. Bezouška was the first/corresponding author or co-author, and heard additional witnesses. In addition to the two above mentioned cases the Commission found scientific misconduct by Prof. Bezouška in at least three other cases. Unfortunately, before the completion of the investigation of the

Commission the verification of the data of Prof. Bezouška (which other authors failed to confirm) was not available.

The Ethics Committee acknowledged, in particular, three female students working in an Institute of Microbiology AV ČR, who first brought to light the evidence of possible scientific misconduct. The investigation also showed how important it is that manuscripts before submission are thoroughly discussed by the whole team of workers and by cooperating teams. During these discussions the primary data should be available in order to prevent the publication of erroneous results. The Commission recommends that all the contributions of Prof. K. Bezouška should now be critically reviewed mainly with respect to the primary data and its interpretation.

The Ethics Committee expressed the belief that this case of "bad behavior in science" was a unique event in Czech science and will not happen again.

Contact person: Coordinator of the Ethical Commission, Assoc. Prof. Dr. Jan Černý jan.cerny@natur.cuni.cz.

Translation from Czech language has been provided by the authors of this paper.

Full version in Czech language available: http://www.biomed.cas.cz/mbu/doc/VyjadreniEK.PDF (Institute of Microbiology, Prague, Czech Republic) accessed on 15 January 2014 and http://www.natur.cuni.cz/fakulta/media/tiskove-zpravy/2013/tiskova-zprava-1 (Charles University in Prague, Czech Republic) accessed on 15 January 2014.

© 2014 by the authors; licensee MDPI, Basel, Switzerland. This article is an open access article distributed under the terms and conditions of the Creative Commons Attribution license (http://creativecommons.org/licenses/by/3.0/).
